# 

**Published:** 1898-07-02

**Authors:** 


**Notices of Births, Deaths, and Marriages** are inserted in this column at a charge of 2s. 6d. for an announcement not exceeding four lines.

### NOTICE TO CORRESPONDENTS.

All MS., Letters, Books for Review, and other matters intended for the Editor should be addressed The Editor, "The Hospital," The Hospital Building, 28 & 29, Southampton Street, Strand, W.C.

The Editor cannot undertake to return rejected MS., even when accompanied by stamped directed envelope.

**Notice to Advertisers and Subscribers.**

All Advertisements, Orders for Copies of The Hospital, and Business Communications should be addressed to **THE MANAGER, (Not to The Editor),** The Hospital, The Hospital Building, 28 & 29, Southampton Street, Strand, London, W.C.

The Cost of Subscription, post free, is as follows:—Per week, 2½d. per quarter, 2s. 8d.; per half-year, 5s. 3d.; per annum, 10s. 6d. Foreign:—Per annum, 15s. 2d.; payable, in all cases, in advance.

**NOTICE.—Vol. XXIII. of "The Hospital" (October, 1897, to March, 1898), in handsome cloth binding, is now on sale at the Office of this Journal, price 7s. 6d. Cases for binding the Numbers contained in this Volume, 1s. 6d. Index to Vol. XXIII., 2½d., post free.**

**The Monthly Part for July is now ready. Price 6d., by post 8d.**

### BOOKS RECEIVED.

BAILLIERE, TINDALL, AND COX.

"Outlines of Practical Surgery." By Walter G. Spencer, M.B., M.S., F.R.C.S.

YOUNG J. PENTLAND.

"Handbook of Obstetric Nursing." By Francis W. N. Haultain, M.D., F.R.C.P.Edin., and James Haig Ferguson, M.D., F.R.C.P.Ed.

FERRIS AND CO.

"Pocket Notes on the New British Pharmacopœia, 1898.

PERKINS, BACON, AND CO.

"Boron Food Preservatives."

J. WRIGHT AND CO.

"Baby Feeding."

### SCRAPS AND GLEANINGS.

THE Local Government Board have signified their sanction to a loan for carrying out at Exeter the septic tank system of sewage treatment.

A DONATION of £250 has been contributed to the Dental Hospital of London by the trustees of Smith's (Kensington Estate) Charity.

AN anonymous donor, "G. R.," has sent £1,000 to St. Thomas's Hospital for the endowment of a medical bed.

THE Lord-Lieutenant of Surrey, Viscount Midleton, on Saturday last opened a Cottage Hospital and Nurses' Home at Haslemere, presented to the town by Mr. J. W. Penfold and his sisters in honour of the Diamond Jubilee.

AFTER the morning service last Sunday at the Foundling Hospital, H.R.H. the Duke of Cambridge presented eight young women and six men, formerly educated at the institution, each with a purse containing five guineas, as a reward for filling situations creditably. The girls had remained four years in domestic service, and two young men had completed a seven years' apprenticeship, and two had served an equal time in the army.

### THE STUDENT OF MEDICINE.

### APPOINTMENTS.

A. J. Bennett, appointed Assistant Medical Officer for the Gordon Road Workhouse and Children's Homes at Heaton Road of the Parish of St. Giles, Camberwell. J. F. Bill, M.B.Lond., M.R.C.S., L.R.C.P., appointed Senior Resident Medical Officer to the London Temperance Hospital. W. F. V. Bonney, M.B., B.S., M.R.C.S., L.R.C.P., appointed Resident Medical Officer to the Chelsea Hospital for Women. W. A. Clark, M.D., M.R.C.S., L.R.C.P., appointed Clinical Assistant to the Chelsea Hospital for Women. E. B. Denny, L.R.C.P.Edin., L.R.C.S.I., appointed Medical Officer for the Hainton District of the Louth Union. P. Fraser, M.D., appointed Medical Officer of Health to the Llanrwst Urban District Council. T. Gibson, M.B., appointed Assistant Medical Officer to the Infirmary of the Parish of St. Giles, Camberwell. Dr. J. Gordon, appointed Medical Officer for the South Hougham District of the Dover Union. G. H. Hunter, M.R.C.S.Eng., appointed Medical Officer for the Haughley District of the Stow Union. Dr. Laurie, appointed Medical Officer for the Moreton District of the Newton Union. F. Lewis, appointed Medical Officer for the Fifth District of the Steyning Union. J. K. Lewis, M.R.C.S.Eng., appointed Medical Officer for the Rewe District of the St. Thomas Union. P. Rendall, M.D., M.R.C. S.Eng., appointed Honorary Surgeon to the Epsom and Ewell Cottage Hospital. J. W. Hart, M.B., C.M.Edin., appointed Government Medical Officer and Vaccinator for the Gunning District. S. J. Hunt, L.R.C.P.-Edin., M.R.C.S.Eng., appointed Government Pathologist for Queensland. R. W. Lethbridge, M.B., appointed Acting Medical Superintendent to the Ballarat Lunatic Asylum, Victoria. W. G. Maddox, L.R.C.S.Eng., appointed Honorary Consulting Surgeon to the Launceston Hospital, Tasmania. H. W. Morley, M.R.C S.Eng., L.R.C.P.Lond., appointed Resident Medical Officer at the Infirmary of the Portsea Island Union. W. L. Mullen, M.D., appointed Acting Medical Superintendent to the Sunbury Lunatic Asylum, Victoria. A. R. Oliver, M.B., C.M., appointed Extra Dispensary Surgeon to the Glasgow Central Dispensary. J. M'I. Pardey, M.B., B.Ch.Melb., appointed Honorary Consulting Surgeon to the Launceston Hospital, Tasmania. C. J. Powell, L.R.C.S.Irel., L.R.C.P.Irel., L.M., Rotunda Hospital, appointed Visiting Physician to the North Dublin Union Hospital. A. F. Seicome, L.R.C.P., L.R.C.S.-Edin., L.F.P.S.Glasg., appointed Assistant House Surgeon to the General Infirmary, Chester. R. B. Sidebottom, L.R.C.P., L.R.C.S.Edin., appointed Medical Officer for the Whitfield District of the Glossop Union. E. Sinclair, M.D., Ch.M.Glasg., appointed Inspector-General of the Insane of N.S.W. J. F. Tabb, M.R.C.S.Eng., L.S.A., appointed Medical Officer for the East District of the Greenwich Union. J. L. Thompson, M.B., appointed Acting Medical Superintendent to the Ararat Lunatic Asylum, Victoria. H. H. Thomson, M.B., C.M., appointed Extra Dispensary Physician to the Glasgow Central Dispensary. May Thorne, L.S.A Lond., M.D.Brux., appointed Assistant Anæsthetist to the New Hospital for Women. G. N. Turner, M.B., C.M., appointed Physician to the Glasgow Central Dispensary. W. D. Wiggins, M.R.C.S., L.R.C.P.Lond., appointed Assistant Medical Officer to the Greenwich Union Workhouse and Infirmary. D. E. Williams, L.R.C.P.&S.Irel., appointed Health Officer for Bunbury, Western Australia. J. D. Williams, M.D., B.Sc.Edin., appointed Honorary Consulting Gynæcologist to the Cardiff Provident Dispensary, Cardiff. T. J. Wood, M.D.Lond., M.R.C.S., L.R.C.P., appointed Assistant Surgeon to the Bradford Royal Infirmary. A. Young, B.Sc., M.B., C.M., appointed Surgeon to the Glasgow Central Dispensary.

THE JOURNAL OF TROPICAL MEDICINE.

A Monthly Journal dealing with the DISEASES of WARM CLIMATES will be issued August 1st, 1898. 17s. annually, post free. Intending Contributors and Subscribers to communicate with:—

The Editors—

JAMES CANTLIE, M.B., F.R.C.S.;

W. J. SIMPSON, M.D., M.R.C.P.;

Care of Messrs. John Bale, Sons, and Danielsson, Ltd., 85-89, Great Titchfield Street, London, W.

